# ﻿Plastid genome variation with phylogenetic implications for the *Helichrysum*-*Anaphalis*-*Pseudognaphalium* (HAP) of the tribe Gnaphalieae (Asteraceae)

**DOI:** 10.3897/phytokeys.262.153120

**Published:** 2025-09-12

**Authors:** Jue Wu, Ying Meng, Ding-Wei Huang, Zi-Han Liu, Xiao-Lin Lao, Jun Wen, Ze-Long Nie

**Affiliations:** 1 Hunan Provincial Key Laboratory of Ecological Conservation and Sustainable Utilization of Wulingshan Resources, College of Biology and Environmental Sciences, Jishou University, Jishou, Hunan, 416000, China Jishou University Jishou China; 2 Department of Botany, National Museum of Natural History, Smithsonian Institution, Washington, DC, 20013-7012, USA National Museum of Natural History, Smithsonian Institution Washington United States of America

**Keywords:** Chloroplast genome, Gnaphalieae, HAP clade, phylogenomics

## Abstract

As the largest lineage in the tribe Gnaphalieae of Asteraceae, phylogenetic relationships within the *Helichrysum*-*Anaphalis*-*Pseudognaphalium* (HAP) remain poorly resolved. In this study we sequenced chloroplast genomes from 28 representative species from the HAP clade, performing comparative and phylogenetic analyses. Their chloroplast genomes ranged from 151,69 to 153,603 bp and possessed a typical quadripartite structure encoding 131–134 genes, including 87–89 protein-coding genes, 36–37 tRNA genes and 8 rRNA genes. The plastome data recognized the HAP into an early-diverging lineage and a large core clade. The latter was further separated into two subclades, namely core I and core II. Relatively small genomes with more insertion and fewer deletions were found in the early HAP lineage and *Anaphalis* taxa from core I. The small chloroplast genomes of the early lineage in the HAP clade potentially linked to their dry environmental adaptation in South Africa. The *trn*T-GGU gene was presented as pseudogenes in the early lineage and a subclade of core II but completely lost in the whole core I. As a distinct lineage within the HAP clade, *Pseudognaphalium* was characterized by the presence of an *ycf*15 pseudogene with only 54 bp and the lack of hexanucleotide repetitions. The findings in this study enhance our understanding of the internal structure of chloroplast genomes in the HAP clade and provide valuable insights on the phylogenetics and plastome evolution of this clade.

## ﻿Introduction

Gnaphalieae is a tribe of the sunflower family (Asteraceae or Compositae) with about 178 genera and 2100 species widely spread across all the landmasses except for the Antarctic (Smissen et al. 2020). Members of the tribe are primarily found in the mountainous regions of the Southern Hemisphere, with notable concentrations in southern Africa, Oceania, and the Americas (Smissen et al. 2011; Nie et al. 2016). Fewer taxa occur in the Northern Hemisphere, but with a significant representation there (Nie et al. 2016). Both ribosomal nuclear and plastid data indicated that the tribe was subdivided into two subtribes: a largely African-endemic basal lineage of Relhaniinae and a big core group of Gnaphaliinae (Smissen et al. 2020). Three major clades were recognized with the crown radiation group, i.e., HAP, FLAG, and Australasian (AUS) clades (Smissen et al. 2011, 2020; Galbany-Casals et al. 2014; Nie et al. 2016).

The HAP clade, including *Helichrysum* Mill., *Achyrocline* (Less.) DC., *Anaphalis* DC., *Pseudognaphalium* Kirp., and other small genera, is the most diversified lineage within the tribe. It includes more than 800 species, more than one-third species found within the tribe (Bergh and Linder 2009; Ward et al. 2009; Smissen et al. 2011, 2020; Galbany-Casals et al. 2014; Nie et al. 2016). Given the high species diversity of *Helichrysum* and morphological heterogeneity within the genus, elucidating its taxonomic and phylogenetic relationships, previously based on morphological characters and limited molecular fragments, remains problematic (Anderberg 1991; Bayer et al. 2002; Smissen et al. 2020). Phylogenetic analyses also suggested that *Helichrysum* was paraphyletic with other genera nested within it (Bergh and Linder 2009; Ward et al. 2009; Smissen et al. 2011, 2020; Galbany-Casals et al. 2014; Nie et al. 2016; Castillo et al. 2024).

Although a large number of studies have been conducted on the HAP group, only limited information on phylogenetic relationships has been available due to insufficient molecular markers, resulting in a poor understanding of the relationships within the clade (Galbany-Casals et al. 2009, 2014; Blanco-Gavaldà et al. 2023). When relying on a limited number of sequences from nuclear genes or plastid molecular markers, the resulting phylogenetic trees often exhibit poor signal in addition to gene introgression and/or incomplete lineage sorting (Smissen et al. 2011; Nie et al. 2013, 2016; Bergh et al. 2015; Lao et al. 2024).

Chloroplast genomes are usually highly conserved regarding gene content and organization within a specific lineage but provide irreplaceable information for phylogenetic and evolutionary studies among taxa (Lao et al. 2024; Xu et al. 2024). For example, gene loss, structural rearrangement, and inversions occur across different taxa and are thought to be key to understanding the origin and evolutionary history of plants (Kumar et al. 2014; Gonçalves et al. 2020). With advancements in sequencing technology, there has been extensive research on chloroplast genomes in many plants (Gonçalves et al. 2020; Lao et al. 2024). Although Lao et al. (2024) had recently utilized plastids for phylogenetic and comparative genomic analyses for the Gnaphalieae, no research has focused on the chloroplast genome structure and comparative genomics of the HAP clade.

Therefore, this study aims to assemble and compare the chloroplast genome content and structure of the HAP members, using plastome genomes to provide a well-supported phylogeny for this group. First, we assembled the chloroplast genomes, elucidated their basic structure, analyzed their genetic features, and explored the composition of chloroplast genes through comparison among different species. Subsequently, we inferred phylogenetic relationships and explored possible implications of plastome variation within the group. Overall, our results will improve our knowledge of chloroplast genome variation, genetic diversity, and phylogenetic relationships within the HAP clade.

## ﻿Material and methods

### ﻿Plant material, DNA extraction and Illumina sequencing

We sampled 42 chloroplast genome sequences, comprising 28 species from the HAP clade (Suppl. material 1: table S1). The sampling covered a variety of representatives from the HAP clade, including *Helichrysum*, *Anaphalis*, *Pseudognaphalium*, and *Achyrocline* (Suppl. material 1: table S1). All the samples were collected from natural habitats worldwide and newly sequenced except for 12 taxa obtained from Lao et al. (2024) (Suppl. material 1: table S1).

Following the CTAB protocol for DNA extraction from Lodhi et al. (1994), we extracted total DNAs following a quantification using a Qubit 4.0 fluorometer with a high-sensitivity kit (Thermo Fisher Scientific, Waltham, MA, USA). The DNA was then sheared to a target size of approximately 300–500 bp via sonication (QSonica Q800R3, Newtown, CT, USA). According to the manufacturer’s protocol, DNA libraries were prepared using the NEBNext Ultra II DNA Prep Kit (New England Biolabs, Ipswich, MA, USA). Genomic sequencing was performed on an Illumina HiSeq 4000 platform with 150 bp paired-end reads.

### ﻿Genome assembly and annotation

The GetOrganelle 1.7.7 pipeline (Jin et al. 2020) was employed to assemble the complete plastomes of all taxa with default parameters. Assembled sequences were visually adjusted in Bandage 0.8.1 (Wick et al. 2015). GeSeq (Tillich et al. 2017) was used to annotate each assembled chloroplast genome. The annotations were cross-validated using other tools, including the standalone plastid annotation pipeline Chloe 0.1.0 (https://chloe.plastid.org/annotate.html) and tRNAscan-SE 2.0.7 within GeSeq. OGDRAW (Greiner et al. 2019) was utilized to visualize the annotated chloroplast genomes. The results were meticulously reviewed and manually adjusted to ensure accuracy and consistency using Geneious 9.0.2 (Kearse et al. 2012), referencing the relevant taxa within the HAP clade. Additionally, we extracted intergenic regions from these 28 species to perform statistical and comparative analyses on insertions and deletions.

### ﻿Phylogenetic analyses

A total of 42 chloroplast genome sequences were included in the phylogenetic analysis. *Calendula arvensis* was selected as outgroup based on results from Nie et al. (2016) and Lao et al. (2024). The complete plastome sequences, including protein-coding genes and non-coding regions, were used for phylogenetic reconstruction. Sequences were aligned using MAFFT 7.427 (Katoh and Standley 2013). Phylogenetic relationships were reconstructed using maximum likelihood (ML) and Bayesian inference (BI) methods. RAxML 8.2.10 (Stamatakis 2006) was employed for the ML analysis with the nucleotide substitution model as determined by MrModeltest 2.4 (Nylander 2004). Rapid bootstrap analyses with 1000 replicates were performed to assess the statistical bootstrap support (BS) for the inferred phylogenetic relationships (Stamatakis 2014).

A Bayesian tree was generated using MrBayes 3.2.7 (Ronquist et al. 2012) with the best-fit model (GTR+I+G) as used in ML analysis. Four Metropolis-coupled Markov chain Monte Carlo simulations were conducted for 20,000,000 generations, with trees sampled every 1,000. Trees retrieved before reaching the likelihood convergence (ca. 4,000 trees) were discarded as burn-ins. The remaining trees were used to build a 50% majority-rule consensus tree with each node’s posterior probabilities (PP).

### ﻿Chloroplast genome comparison

MISA 2.1 (https://webblast.ipk-gatersleben.de/misa/) was employed to identify repetitive sequences and simple sequence repeats (SSRs) within the chloroplast genomes with mono-, di-, tri-, tetra-, penta-, and hexanucleotide repetitions, the respective thresholds were set as 10, 6, 5, 3, 3, and 3, respectively (Beier et al. 2017). Additionally, REPuter (https://bibiserv.cebitec.uni-bielefeld.de/reputer) was utilized to detect various repeated sequences, including forward, palindromic, reverse, and complementary repetitions. During this process, a hamming distance of three was maintained, and the minimum repeat size was established at thirty base pairs (Kurtz et al. 2001).

Chloroplast were compared using mVISTA (Mayor et al. 2000; https://genome.lbl.gov/vista/mvista/submit.shtml) in Shuffle-LAGAN mode (Frazer et al. 2004). We analyzed the boundaries of the large single copy (LSC), inverted repeat (IR), and small single copy (SSC) regions of each chloroplast genome using CPJSdraw 1.0.1 (Li et al. 2023). We also conducted a comprehensive genome comparison using the MAUVE plugin in Geneious 9.0.2 (Kearse et al. 2012). To identify genetic variations among species, we computed nucleotide diversity using DnaSP (Rozas et al. 2017), employing a sliding window analysis with an 800 bp window length and a 200 bp step size.

Codon usage bias is the preferential or non-random use of synonymous codons, commonly associated with gene expression and nucleotide composition. The relative synonymous codon usage (RSCU) values were determined by comparing the frequency of a specific codon to the average frequency of all codons encoding the same amino acid, which can be acquired through JSHYCloud (http://cloud.genepioneer.com:9929). We performed RSCU analysis on 29 chloroplast genome sequences and utilized the “heatmap” function in Rstudio (RStudio Team 2020) to generate clustering heatmaps based on the RSCU data.

The rates of non-synonymous substitutions (Ka) and synonymous substitutions (Ks) can be used to determine whether gene sequences have had selective pressure (Nguyen et al. 2015). The Ka/Ks ratio indicates the degree of gene divergence and the type of selection pressure: positive selection (Ka/Ks > 1), purifying selection (Ka/Ks < 1, particularly if less than 0.5), or neutral selection (Ka/Ks = 1) (Kimura 1989). We extracted the shared genes among species and aligned each CDS using MAFFT (Katoh and Standley 2013). Subsequently, the Ka/Ks ratios were calculated with the KaKs_Calculator in ParaAT 2.0 (Zhang et al. 2006) using the command “ParaAT.pl -c 11 -h homologs.txt -n CDS -a PEP -p proc -o OUT -k -f axt -m mafft -v”.

## ﻿Results

### ﻿Chloroplast genome content and feature

**Figure 1.**
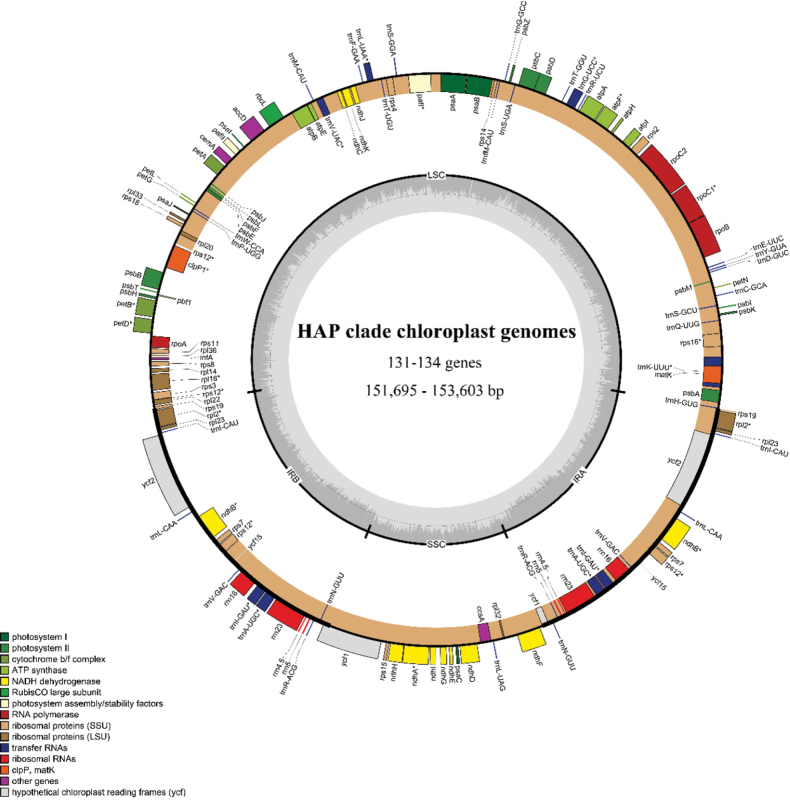
Structure and characteristics of the complete chloroplast genomes from the HAP clade. Genes outside the circle indicate counterclockwise transcription, and genes inside the clockwise transcription. The thick black line on the outer circle represents the two IR regions. The GC content is the dark gray area within the ring.

**Figure 2.**
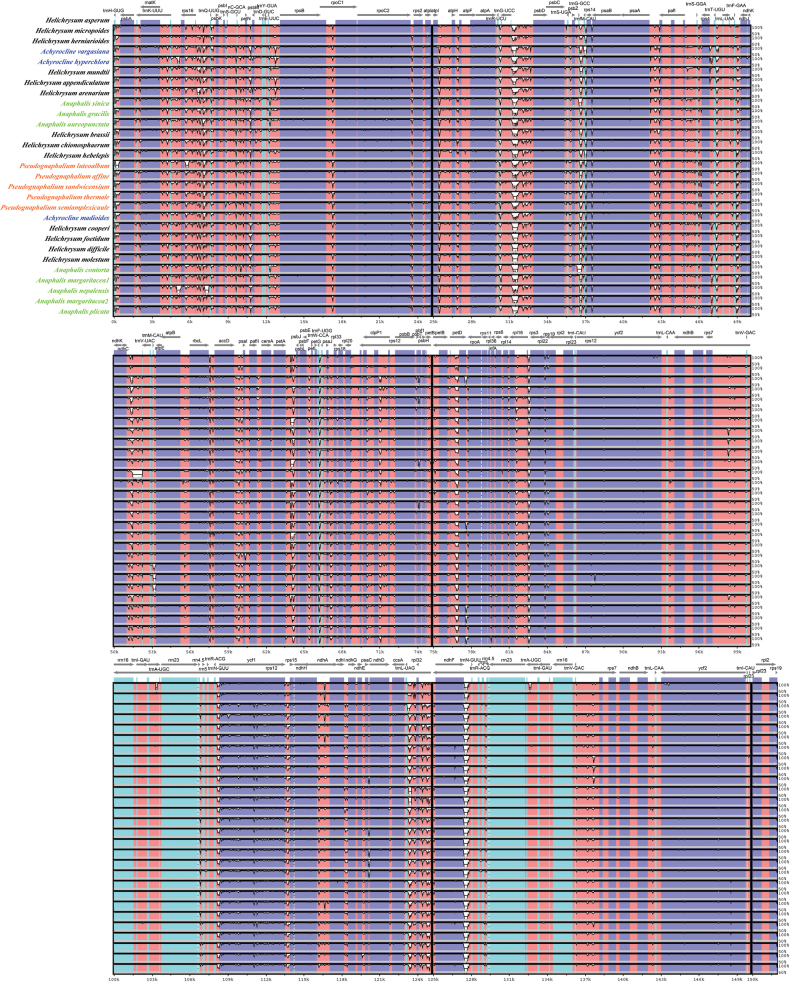
Visualization of genome alignment of the 29 chloroplast genomes from the HAP. Gray arrows and thick black lines above the alignment indicate the direction of the gene. Purple bars represent exons, blue bars represent untranslated regions (UTRs), pink bars represent conserved non-coding sequences (CNS), and gray bars represent mRNA. The Y-axis shows different species names, and sequence similarity of aligned regions is displayed as horizontal bars.

All the chloroplast genomes of the HAP clade displayed a typical structure made up of two inverted repeats (IRa/b) that split the whole genome into a large single-copy (LSC) and a small single-copy (SSC) region (Fig. 1). There was no significant difference in the size, gene, and genome structure among the studied chloroplast genomes (Fig. 2; Suppl. material 1: fig. S1; Tables 1, 2). The MAUVE results indicated that no gene recombination or rearrangement was detected within the analyzed sequences (Suppl. material 1: fig. S2). The overall GC content in the HAP clade ranged from 37.1% to 37.2%, with the IR regions showing higher GC content than the LSC and SSC regions (Table 2).

**Table 1.** Genes identified in the chloroplast genomes of HAP clade.

Category	Group of genes	Name of genes
Photosynthesis	Photosystem I	*psa*A, *psa*B, *psa*C, *psa*I, *psa*J
	Photosystem II	*psb*A, *psb*B, *psb*C, *psb*D, *psb*E, *psb*F, *psb*H, *psb*I, *psb*J, *psb*K, *psb*L, *psb*M, *psb*T, *psb*Z
	NADH dehydrogenase	*ndh*A^a^, *ndh*B^a^(×2), *ndh*C, *ndh*D, *ndh*E, *ndh*F, *ndh*G, *ndh*H, *ndh*I, *ndh*J, *ndh*K
	Cytochrome b/f complex	*pet*A, *pet*B^a^, *pet*D^a^, *pet*G, *pet*L, *pet*N
	Polymerase associated factor	*paf*I^b^, *paf*II
	Photosystem biogenesis factor	*pbf*1
	ATP synthase	*atp*A, *atp*B, *atp*E, *atp*F^a^, *atp*H, *atp*I
Self-replication	Ribosomal proteins (SSU)	*rps*2, *rps*3, *rps*4, *rps*7(×2), *rps*8, *rps*11, *rps*12^b^(×2), *rps*14, *rps*15, *rps*16^a^, *rps*18, *rps*19(×2)
	Ribosomal proteins (LSU)	*rpl*2^a^(×2), *rpl*14, *rpl*16^a^, *rpl*20, *rpl*22, *rpl*23(×2), *rpl*32, *rpl*33, *rpl*36
	Ribosomal RNAs	*rrn*4.5(×2), *rrn*5(×2), *rrn*16(×2), *rrn*23(×2)
	Transfer RNAs	*trn*A-UGC^a^(×2), *trn*C-GCA, *trn*D-GUC, *trn*E-UUC, *trn*F-GAA, *trn*G-UCC^a^, *trn*G-GCC^a^, *trn*H-GUG, *trn*I-CAU(×2)/GAU^a^(×2), *trn*K-UUU^a^, *trn*L-UAG/UAA^a^/CAA(×2), *trn*M-CAU, *trnf*M-CAU, *trn*N-GUU(×2), *trn*P-UGG, *trn*Q-UUG, *trn*R-ACG(×2)/UCU, *trn*S-GCU/GGA/UGA, #*trn*T-GGU/UGU, *trn*V-GAC(×2)/UAC^a^, *trn*W-CCA, *trn*Y-GUA
Other genes	DNA-dependent RNA polymerase	*rpo*A, *rpo*B, *rpo*C1^a^, *rpo*C2
	Maturase	*mat*K
	Protease	*clp*P1^b^
	Envelope membrane protein	*cem*A
	Subunit acetyl-CoA-carboxylase	*acc*D
	c-Type cytochrome synthesis gene	*ccs*A
	Subunit of Rubisco	*rbc*L
	Translation initiation factor	*inf*A
Genes of unknown function	Conserved open reading frames	*ycf*1(×2)^h^, *ycf*2(×2), #*ycf*15(×2)

a represents a gene with one intron; b represents a gene with two introns; (×2) represents Number of copies of multi-copy genes; # represents 12 species deficient in the trnT-GGU gene, including *Helichrysum appendiculatum*, *H. arenarium*, *H. asperum*, *H. herniarioides*, *H. micropoides*, *H. mundtii*, *Achyrocline vargasiana*, *A. hyperchlora*, *Anaphalis sinica*, *A. aureopunctata*, *A. gracilis*, *A. contorta*.

**Table 2.** Comparison of the general features of the chloroplast genomes from HAP clade.

Taxa	Length (bp)				Gene number				GC (%)
	Total	LSC	SSC	IR	gene	CDS	rRNA	tRNA	
*Achyrocline hyperchlora*	152944	84683	18651	24805	131	87	8	36	37.1
*Achyrocline madioides*	153455	85197	18502	24878	132	87	8	37	37.1
*Achyrocline vargasiana*	153603	85472	18405	24863	131	87	8	36	37.1
*Anaphalis aureopunctata*	152786	84592	18510	24842	131	87	8	36	37.1
*Anaphalis contorta*	153155	84892	18523	24870	131	87	8	36	37.1
*Anaphalis gracilis*	152657	84442	18533	24841	131	87	8	36	37.1
*Anaphalis margaritacea* 1	153359	85093	18498	24884	132	87	8	37	37.1
*Anaphalis margaritacea* 2	153239	84959	18512	24884	132	87	8	37	37.1
*Anaphalis nepalensis*	152841	84598	18475	24884	132	87	8	37	37.1
*Anaphalis plicata*	153307	85035	18506	24883	132	87	8	37	37.1
*Anaphalis sinica*	152796	84601	18517	24839	131	87	8	36	37.1
*Helichrysum appendiculatum*	153199	84930	18527	24871	131	87	8	36	37.1
*Helichrysum arenarium*	152503	84318	18467	24859	131	87	8	36	37.2
*Helichrysum asperum*	151923	84063	19284	24288	130	86	8	36	37.2
*Helichrysum brassii*	152851	84827	18264	24880	132	87	8	37	37.1
*Helichrysum chionosphaerum*	152231	84185	18294	24876	132	87	8	37	37.1
*Helichrysum cooperi*	153164	84918	18504	24871	132	87	8	37	37.1
*Helichrysum difficile*	153383	85127	18514	24871	132	87	8	37	37.1
*Helichrysum foetidum*	153249	85008	18499	24871	132	87	8	37	37.1
*Helichrysum hebelepis*	153377	85167	18466	24872	132	87	8	37	37.1
*Helichrysum herniarioides*	151836	83524	18996	24658	131	87	8	36	37.2
*Helichrysum micropoides*	151695	83637	18952	24553	131	87	8	36	37.2
*Helichrysum molestum*	153308	85071	18495	24871	132	87	8	37	37.1
*Helichrysum mundtii*	153220	84930	18536	24877	131	87	8	36	37.1
*Pseudognaphalium affine*	153049	84809	18500	24870	134	89	8	37	37.1
*Pseudognaphalium luteoalbum*	153179	84953	18486	24870	134	89	8	37	37.1
*Pseudognaphalium sandwicensium*	153410	85170	18500	24870	134	89	8	37	37.1
*Pseudognaphalium semiamplexicaule*	153464	85207	18501	24878	134	89	8	37	37.1
*Pseudognaphalium thermal*	153236	84971	18511	24877	134	89	8	37	37.1

The HAP plastomes contained 131–134 genes, including 87–89 protein coding genes, 36–37 tRNA genes and 8 rRNA genes (Tables 1, 2). The *ycf*15 gene was presented within all the *Pseudognaphalium* species but missing for the others within the HAP clade. All members of subclade II had the *trn*T-GGU gene except for *Anaphalis contorta* (Tables 1, 2).

### ﻿Phylogenetic relationships within HAP

**Figure 3.**
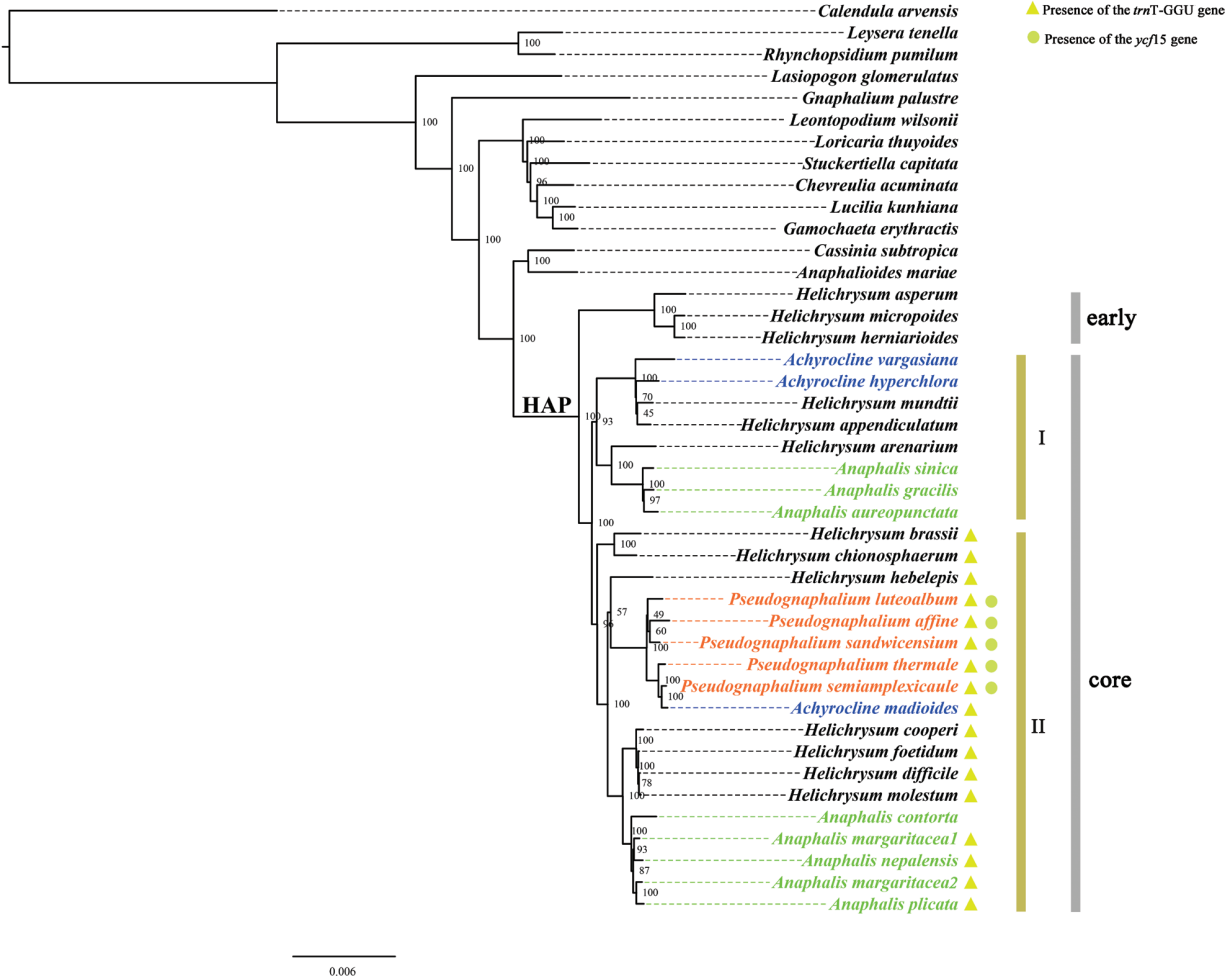
A maximum likelihood tree based on 42 complete chloroplast sequences from the HAP clade and relatives of Gnaphalieae. Numbers above branches indicate bootstrap values from 1000 replicates. Species names with colors represent different genera.

The ML and Bayesian analysis yielded similar topologies, with most deep nodes showing strong support, evidenced by BS > 90% and PP > 0.95 (Fig. 3; Suppl. material 1: fig. S3). The HAP clade had the closest relationship to the Australian clade, consisting of *Cassinia subtropica* and *Anaphalioides mariae* (Fig. 3). *Helichrysum* was not monophyletic, with taxa from *Anaphalis*, *Achyrocline*, and *Pseudognaphalium* nested within it (Fig. 3). The HAP was separated into an early diverged clade constituted by species from southern Africa, followed by a main clade containing two subclades, core I and II (Fig. 3). The genus *Pseudognaphalium* was recovered as paraphyletic with *Achyrocline madioides* nested in (BS = 76%, PP = 0.99, Fig. 3; Suppl. material 1: fig. S3); the remaining two members of *Achyrocline* sampled were nested in subclade I (BS = 94%, PP = 1) together with *Helichrysum mundii* (from Africa) and *H. appendiculatum* (Fig. 3; Suppl. material 1: fig. S3). Finally, members of *Anaphalis* were clustered into two groups placed in subclades I and II (Fig. 3).

### ﻿Genome structure

The total lengths of chloroplast genomes ranged from 151, 695 to 153, 603 bp (Table 2). The largest chloroplast genome length was found in *Achyrocline vargasiana* and the smallest one was found in *Helichrysum micropoides* (Table 2).

**Figure 4.**
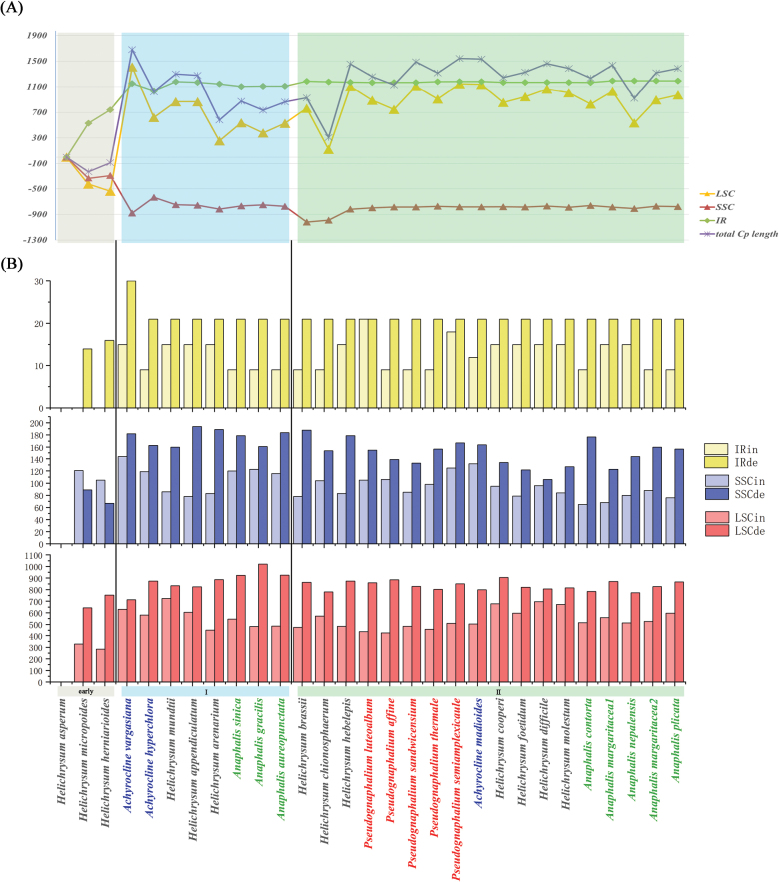
Statistics of chloroplast genome length and number of insertion and deletion fragments. A. Length plastome and subregions (LSC, SSC, IR) comparison; B. Insertion (-in) and deletion (-de) fragments comparison. Colors represent different genera.

The early lineage, represented by *Helichrysum asperum*, *H. herniarioides*, and *H. micropoides*, clearly showed comparatively reduced total lengths (Fig. 4). The intergenic regions included the majority of the indels, which may cause the variation of genome length (Fig. 4). Members of the early lineage usually had shorter lengths of LSC and IR regions, but showed relatively large lengths for the SSC region. Early lineage members also showed no insertions in the IR region and less insertions in the LSC (Fig. 4), contrastingly they showed higher numbers of insertion and less deletions in the SSC region (Fig. 4).

For the IR boundary analysis, a relatively organized gene arrangement was observed at the JLB (junction of IRb and LSC), JSB (junction of IRb and SSC), JSA (junction of IRa and SSC), and JLA (junction of IRa and LSC) boundaries (Suppl. material 1: fig. S1). The *rps*19 gene near the JLB was only 40 bp located completely within the LSC side for *Anaphalis contorta*. The *ycf*1 gene spanned the IRb and SSC regions. In *Helichrysum asperum*, 17 bp of the *ycf*1 gene were located within the IRb, while in *Helichrysum herniarioides* and *Helichrysum micropoides*, 356 bp were situated within the IRb. In the remaining species, the *ycf*1 gene region ranged from 522 to 589 bp within the IRb. However, the *ycf*1 gene located at the JSA boundary crosses the boundary in 10 species. *Helichrysum asperum* lacked the *ycf*1 gene at the JSA boundary (Suppl. material 1: fig. S1).

**Figure 5.**
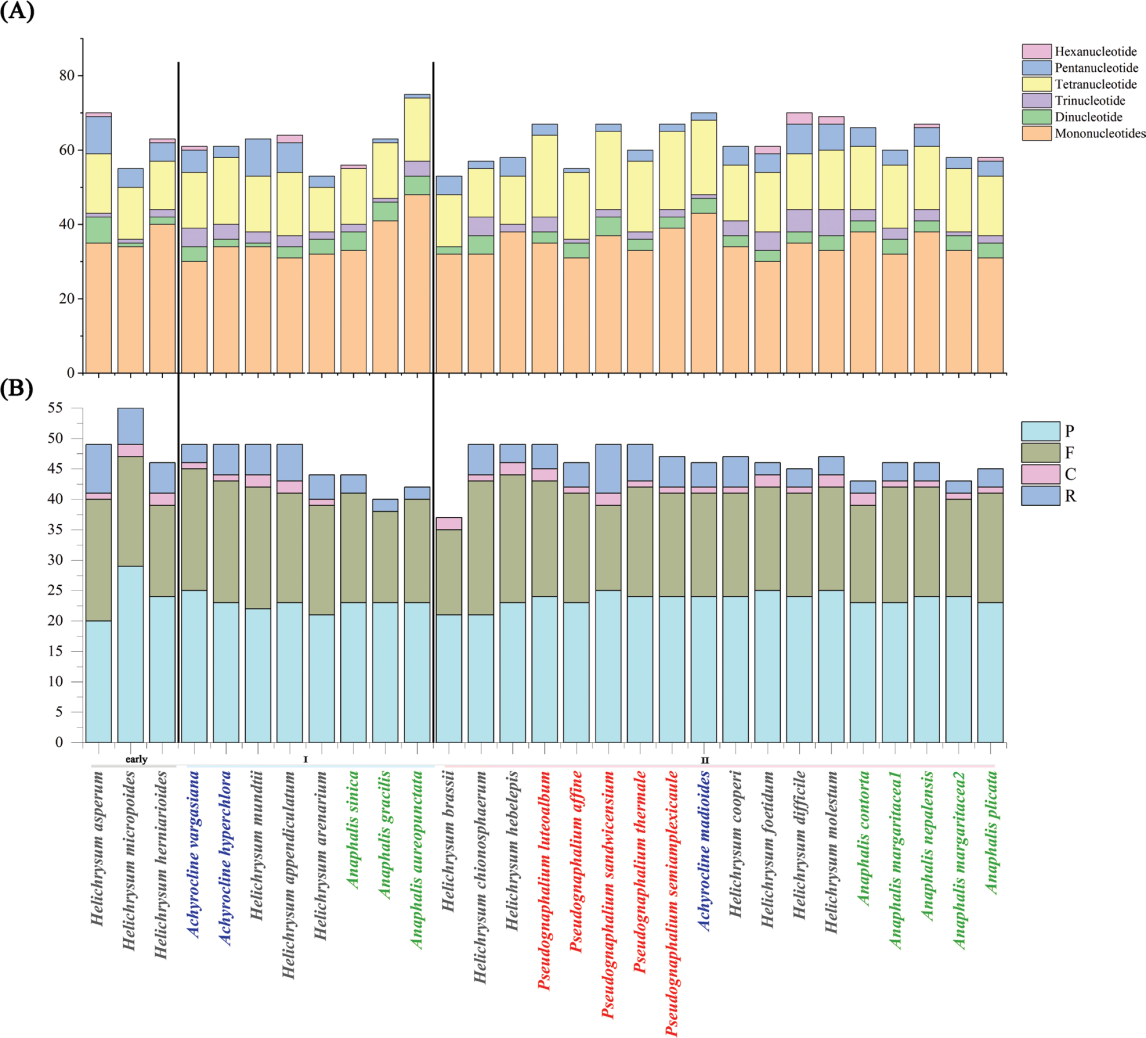
Repeat sequences of the HAP chloroplast genome. A. SSR; B. Dispersed repeats and tandem repeats, Forward (F), Reverse (R), Complement (C), Palindromic (P). Species names with colors represent different genera.

A cumulative count of 1,345 repetitive sequences was identified within the HAP clade, with forward and palindromic repeats displaying greater prevalence compared to complementary and reverse repeats (Fig. 5). A total of 1,808 SSR loci were found across the chloroplast genomes of these 29 sequences (Fig. 5). Among the six types of sequences, single-nucleotide and tetranucleotide sequences were the most abundant, with A and T being the most frequent occurrence within single-nucleotide sequences. *Anaphalis aureopunctata*, *A. sinica*, and *A. gracilis* exhibited a significant reduction in pentanucleotide sequences. Additionally, none of the species within the *Pseudognaphalium* exhibited hexanucleotide sequences.

### ﻿Nucleotide diversity, codon usage, and selection pressure

The nucleotide diversity (Pi) ranged from 0.0000 to 0.02901, with six highly variable regions identified, including *rps*16-*exon*2-*rps*16-*exon*1 (0.01805), *trn*K-*exon*1 (0.0157), and *trn*C-*pet*N (0.0151) from the LSC region, and *ndh*F-*ycf*1 (0.02901), *ycf*1 (0.02811) and *ycf*1-*trn*N (0.02189) from the SSC region (Suppl. material 1: fig. S4). These hypervariable loci demonstrate promising potential to serve as DNA barcoding markers.

A total of 29 codons exhibited codon usage bias with RSCU>1.0 (Suppl. material 1: figs S5, S6). The highest RSCU was 2.0882 for AGA encoding arginine (Arg), while the lowest value was 0.4567 for CGC encoding arginine (Arg). For methionine (Met) and tryptophan (Trp) the RSCU values were both 1 (Suppl. material 1: fig. S6).

**Figure 6.**
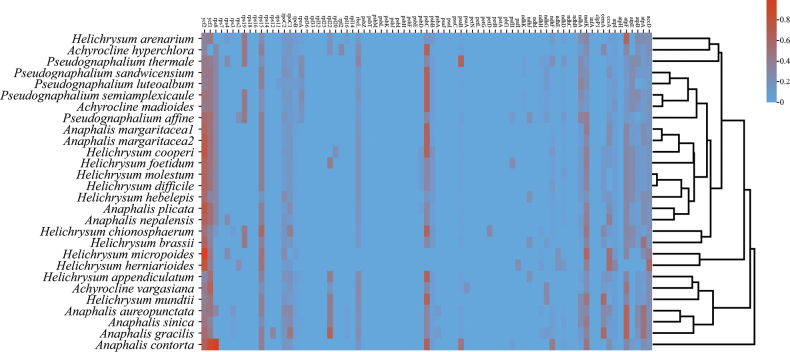
Ka/Ks ratios in protein coding genes of the HAP clade.

The Ka/Ks ratios across examined species ranged from 0 to 0.990664 (Fig. 6). The *rps*8 gene in *Anaphalis contorta* exhibited the maximum value of 0.990664 and the *ycf*2 gene in *Helichrysum micropoides* demonstrated a near-neutral ratio of 0.977114.

## ﻿Discussion

Chloroplast genomes showed a high degree of conservation, probably upheld by various molecular mechanisms, including uniparental inheritance, infrequent plastid fusion, and the existence of an active repair mechanism (Wicke et al. 2011). The genes in all the HAP chloroplast genomes were largely colinear without major rearrangements (Figs 1, 2; Suppl. material 1: figs S1, S2), indicating a relatively conserved chloroplast structure of this group. Similar GC content of the genomes further suggested conserved and similar chloroplast genomes within the HAP (Table 2). The total count and proportional distribution of repetitive sequences exhibited similarities across taxa, revealing conserved characteristics in chloroplast genome structure within the HAP clade.

The HAP clade, including the majority of *Helichrysum* along with *Anaphalis* and *Pseudognaphalium*, and at least part of *Achyrocline*, had been consistently recovered as a well-supported clade within the crown radiation Gnaphalieae (Galbany-Casals et al. 2009, 2014; Ward et al. 2009; Smissen et al. 2011, 2020; Castillo et al. 2024). Two groups were recognized within the HAP clade, including an early-derived lineage and a large clade named as core HAP based on chloroplast data (Fig. 3; Suppl. material 1: fig. S3). This backbone relationship was consistent with previous studies utilizing plastid data (Galbany-Casals et al. 2014; Smissen et al. 2020). However, previous studies provided very low resolution for the core HAP clade from chloroplast sequences (Galbany-Casals et al. 2014; Smissen et al. 2020). ML and Bayesian analyses from plastid genome data revealed well-supported phylogenetic relationships within the core HAP (Fig. 3; Suppl. material 1: fig. S3). Two subclades were recognized within the core HAP: core I, including *Anaphalis* I - *Achyrocline*, and core II, including *Anaphalis* II - *Pseudognaphalium*.

The genus *Anaphalis* was recovered as monophyletic with moderate support sister to a clade comprised by all the Mediterranean, Asian and some of the Macaronesian *Helichrysum* species (Nie et al. 2013; Galbany-Casals et al. 2014). However, the plastome data suggests *Anaphalis* is biphyletic (Fig. 3), similar to the results of Xu et al. (2024). This phylogenetic relationship was further supported by leaf, cypsela, and achene morphologies (Abid and Qaiser 2007; Nie et al. 2013; Xu et al. 2024).

A previous study found that members of *Pseudognaphalium* belonged to a single group constituted two main clades sister to each other without support (Galbany-Casals et al. 2014; Castillo et al. 2022). However, the plastome data indicated that all members of the genus were clustered together as a well-supported lineage that includes *Achyrocline madioides* (Fig. 3). The other two members of the *Achyrocline* sampled were grouped with other *Helichrysum* species, similar to the nrDNA results from Galbany-Casals et al. (2014).

Incongruence between cpDNA and nrDNA had been previously reported for the phyogenetic relationships within the HAP clade (Galbany-Casals et al. 2009, 2010, 2014; Smissen et al. 2011; Blanco-Gavaldà et al. 2025). In previous studies, nuclear data, including both ribosomal and targeted genes, showed a different topology from our chloroplast data with a sizeable basal grade, mainly constituted by many lineages from southern Africa, followed by a main clade containing all members of *Anaphalis*, *Achyrocline*, and *Pseudognaphalium* and several isolated species in polytomy (Galbany-Casals et al. 2014; Blanco-Gavaldà et al. 2023, 2025).

Phylogenetic conflicts were also found from specific groups within the clade when compared with published nuclear data studies. For example, all samples of *Pseudognaphalium* were constituted as a clade along with *Achyrocline madioides* by plastome data (Fig. 3), whereas recent nuclear genomic data supporting this genus was non-monophyletic (Castillo et al. 2022; Blanco-Gavaldà et al. 2023). Two independent main lineages with several less diversified clades were recognized within *Pseudognaphalium* (Blanco-Gavaldà et al. 2023). An allopolyploid origin with past or present hybridization had been hypothesized for many HAP members with evidence from low-copy markers and chromosome data (Smissen et al. 2011), but other factors including incomplete lineage sorting or retention of ancestral polymorphisms can explain the complicate evolutionary relationships with the HAP clade (Ward et al. 2009; Smissen et al. 2011; Galbany-Casals et al. 2014).

Although the plastomes of the HAP are highly conserved, we found that the current phylogenetic relationships were in line with variations in chloroplast genome length, gene loss, and repeat types. The early lineage, represented by *Helichrysum asperum*, *H. herniarioides*, and *H. micropoides*, clearly showed comparatively reduced total lengths (Fig. 4). Members of the early lineage usually had shorter lengths of LSC and IR regions, but showed relatively large lengths for the SSC region. The intergenic regions included the majority of the indels, which may cause the variation of genome length. The early lineage was also characterized by no insertions in the IR region and less insertions in the LSC when compared with the core group (Fig. 4). Furthermore, the *rbc*L and *psb*C genes from this early lineage showed with low Ka/Ks ratios (Fig. 6), which are crucial for photosynthesis, suggesting a potential link between phylogenetic divergence, environmental factors, and gene conservation (Bungard 2004; Yang et al. 2019).

Andrés-Sánchez et al. (2019) demonstrated that the basal species of HAP, including *Helichrysum micropoides*, *H. asperum*, and *H. herniarioides* explored in this study, had a strong association between reduced genome sizes with lower base chromosome numbers and short-lived life-history. Plants with smaller genomes have faster cell division cycles and so higher intrinsic growth rates, probably offering selective advantages in annuals occurring in the arid environments with a short growing-season (Knight et al. 2005; Andrés-Sánchez et al. 2019). Therefore, the relatively small chloroplast genomes of the early lineage in the HAP clade are potentially linked to their dry environmental adaptation in South Africa.

Different plastome genome lengths were also observed for the two lineages of *Anaphalis* (Fig. 3). *Anaphalis* I from core I had shorter genome size than *Anaphalis* II from core II (Fig. 3). The latter also showed less insertion with more deletions from the former. Another feature was that *Anaphalis* I had no complement repeat sequences (Fig. 5). Interestingly, *Anaphalis sinica*, *A. gracilis*, and *A. aureopunctata* from core II exhibited higher Ka/Ks ratios in the *atp*F gene compared to other *Anaphalis* I species (Fig. 6), indicating possible differentiation of selection pressure on genes between these two *Anaphalis* lineages.

We also found that the threonine *trn*T-GGU gene was lacking in the whole core I clade, while presented as pseudogenes in all species in the early lineage and core II with the exception of *Anaphalis contorta* (Fig. 3). However, we found the chloroplast genome sequence of *Anaphalis contorta* showed Ns at the *trn*T-GGU position and should be questioned for its possible poor sequencing quality. The pseudogenization of the *trn*T-GGU gene is commonly found in Asteraceae including many taxa from the tribe Gnaphalieae (Lee et al. 2017; Abdullah et al. 2021). This pseudogenization of *trn*T-GGU was probably associated with an insertion event within the 5′ acceptor stem of the tRNA and did not affect codon usage because a single transfer RNA (*trn*T-UGU) decodes all four threonine codons (Alkatib et al. 2012; Abdullah et al. 2021). In this study, we further detected a complete loss of the *trn*T-GGU in a group of species (core II in Fig. 3). This pseudogenization and gene loss resulted in two types of chloroplast genomes in the HAP clade (Fig. 2).

All *Pseudognaphalium* species had annotated one more gene, *ycf*15 (Fig. 3; Table 1), consistent with those of *P. affine* from the NCBI Genbank (NC_060507 and MN541094). The *ycf*15 found in *Pseudognaphalium* was only 54 bp in length with an early TGA stop codon, much shorter than the *ycf*15 genes found in other angiosperms with about 250 bp in length (Shi et al. 2013). The *ycf*15 is either nonexistent or impaired in other angiosperm species (Shi et al. 2013). The 54 bp of *ycf*15 gene fragment in *Pseudognaphalium* probably is a non-functional segment, its lack of an open reading frame (ORF) suggests that it may be a pseudogene (Wanichthanarak et al. 2023). Interestingly, the lack of repetitions of hexanucleotides in *Pseudognaphalium* species (Fig. 5) further indicated the genus as a distinct group within the HAP clade.

## ﻿Conclusions

In this study, we analyzed the complete chloroplast genomes of 28 species from the HAP clade. Their chloroplast genomes possessed a typical quadripartite and highly conserved structure encoding 131–134 genes, including 87–89 protein-coding genes, 36–37 tRNA genes, and 8 rRNA genes. The genic and IR regions were more conserved than the intergenic and SC regions, respectively. Phylogenetic analyses based on plastomes’ data suggested that the HAP clade was separated into an early-diverged lineage and a large core clade, with the latter further grouped into two subclades. Relatively reduced genome size was found in the early HAP lineage and one of the two *Anaphalis* subclades. The *trn*T-GGU gene was lacking in the whole subclade I while presented in all species in subclade II and the early HAP lineage. Furthermore, an additional *ycf*15 gene was found in all *Pseudognaphalium* species. The findings in this study enhanced our understanding of the structural variation of chloroplast genomes in the HAP clade.

## ﻿Additional information

### Use of AI

No use of AI was reported.

### Author ORCIDs

Jue Wu https://orcid.org/0009-0000-6253-0099

Jun Wen https://orcid.org/0000-0001-6353-522X

### Data availability

All of the data that support the findings of this study are available in the main text or Supplementary Information.

## Supplementary materials

Supplementary material 1 Supplementary table and figures Data type docx Explanation note **table S1**: Taxa with collection information sampled in this study; **figure S1**: Gene order and junction sites for the HAP chloroplast genomes showing connection sites of LSC, IRB, SSC, and IRA; **figure S2**: MAUVE alignment of the HAP chloroplast genomes; **figure S3**: A Bayesian tree based on the chloroplast genome sequences from the HAP and other taxa of Gnaphalieae; **figure S4**: The nucleotide diversity values of the 29 chloroplast genomes from the HAP; **figure S5**: Amino acid frequencies of the chloroplast genomes of the HAP clade; **figure S6**: The heat map of RSCU clustering for the HAP plastomes. https://binary.pensoft.net/file/1414382This dataset is made available under the Open Database License (http://opendatacommons.org/licenses/odbl/1.0/). The Open Database License (ODbL) is a license agreement intended to allow users to freely share, modify, and use this Dataset while maintaining this same freedom for others, provided that the original source and author(s) are credited.Jue Wu, Ying Meng, Ding-Wei Huang, Zi-Han Liu, Xiao-Lin Lao, Jun Wen, Ze-Long Nie
