# Supplementary material for: ﻿Plastid genome variation with phylogenetic implications for the Helichrysum-Anaphalis-Pseudognaphalium (HAP) of the tribe Gnaphalieae (Asteraceae)
Source: PhytoKeys. 2025 Sep 12;262:203–21. doi: 10.3897/phytokeys.262.153120 (PMC12449695; doi:10.3897/phytokeys.262.153120)
Supplement: Supplementary material 1 — Supplementary table and figures [file phytokeys-262-203_article-153120__-s001.docx]

**SUPPLEMENTARY DATA**

Table S1 Taxa with collection information sampled in this study.

| Taxa | | Collection/Source | Locality |  |
| --- | --- | --- | --- | --- |
| *Anaphalis aureopunctata* | Tibet 2478 (KUN, US) | China, Sichuan | | |
| *Anaphalis contorta* | Nie 626 (KUN) | Nepal | |  |
| *Anaphalis gracilis* | Nie 746 (KUN) | China, Yunnan | | |
| *Anaphalis nepalensis* | Tibet 904 (KUN, US) | Nielamu, Tibet | | |
| *Anaphalis margaritacea* 1 | Tibet 1656 (KUN, US) | China, Yunnan | | |
| *Anaphalis margaritacea* 2 | King 11090 (US) | USA, Colorado | | |
| *Anaphalis plicata* | Tibet 156 (KUN, US) | Lasha, Tibet | | |
| *Anaphalis sinica* | Murata 16974 (US) | Japan, Honshiu | | |
| *Achyrocline madioides* | Beck11217 (US) | Bolivia | |  |
| *Achyrocline hyperchlora* | Bastion 1253 (US) | Bolivia | |  |
| *Achyrocline vargasiana* | Schmeda 888 (US) | Paraguay | |  |
| *Helichrysum appendiculatum* | Koekemoer 3438 (US) | South Africa, KwaZulu-Natal | | |
| *Helichrysum brassii* | Chapman 7579 (US) | Malawi | |  |
| *Helichrysum chionosphaerum* | Koekemoer 2318 (US) | KwaZulu-Natal, South Africa | | |
| *Helichrysum difficile* | Carney 13 (US) | South Africa, Gauteng | | |
| *Helichrysum hebelepis* | Koekemoer 3327 (US) | South Africa | | |
| *Helichrysum molestum* | Koekemoer 2230 (US) | Mpumalanga, South Africa | | |
| *Helichrysum mundtii* | Koekemoer 3435 (US) | KwaZulu-Natal, South Africa | | |
| *Helichrysum arenarium* | Barta 2003249 (US) | Europe | |  |
| *Helichrysum asperum* | Koekemoer 3439 (US) | South Africa | | |
| *Helichrysum cooperi* | Kayombo 629 (US) | Tanzania | |  |
| *Helichrysum foetidum* | Koekemoer 3456 (US) | South Africa | | |
| *Helichrysum herniarioides* | Koekemoer 3133 (US) | South Africa | | |
| *Helichrysum micropoides* | Koekemoer 3357 (US) | South Africa | | |
| *Pseudognaphalium luteoalbum* | Robert Merrill 9742 (US) | Victoria, Australia | | |
| *Pseudognaphalium semiamplexicaule* | Fother 11090 (US) | Alta Verapaz, Guatemala | | |
| *Pseudognaphalium thermale* | Calder et al. 15595 (US) | British Columbia, Canada | | |
| *Pseudognaphalium sandwicensium* | Funk12765 (US) | Hawaii | |  |
| *Pseudognaphalium affine* | Tibet 132 (KUN, US) | China, Lasha | | |
| *Anaphalioides mariae* | Lao et al., 2024 |  | | |
| *Cassinia subtropica* | Lao et al., 2024 |  | | |
| *Chevreulia acuminata* | Lao et al., 2024 |  | | |
| *Gamochaeta erythractis* | Lao et al., 2024 |  | | |
| *Gnaphalium palustre* | Lao et al., 2024 |  | | |
| *Lasiopogon glomerulatus* | Lao et al., 2024 |  | | |
| *Leontopodium wilsonii* | Lao et al., 2024 |  | | |
| *Leysera tenella* | Lao et al., 2024 |  | | |
| *Loricaria thuyoides* | Lao et al., 2024 |  | | |
| *Lucilia kunhiana* | Lao et al., 2024 |  | | |
| *Rhynchopsidium pumilum* | Lao et al., 2024 |  | | |
| *Stuckertiella capitata* | Lao et al., 2024 |  | | |


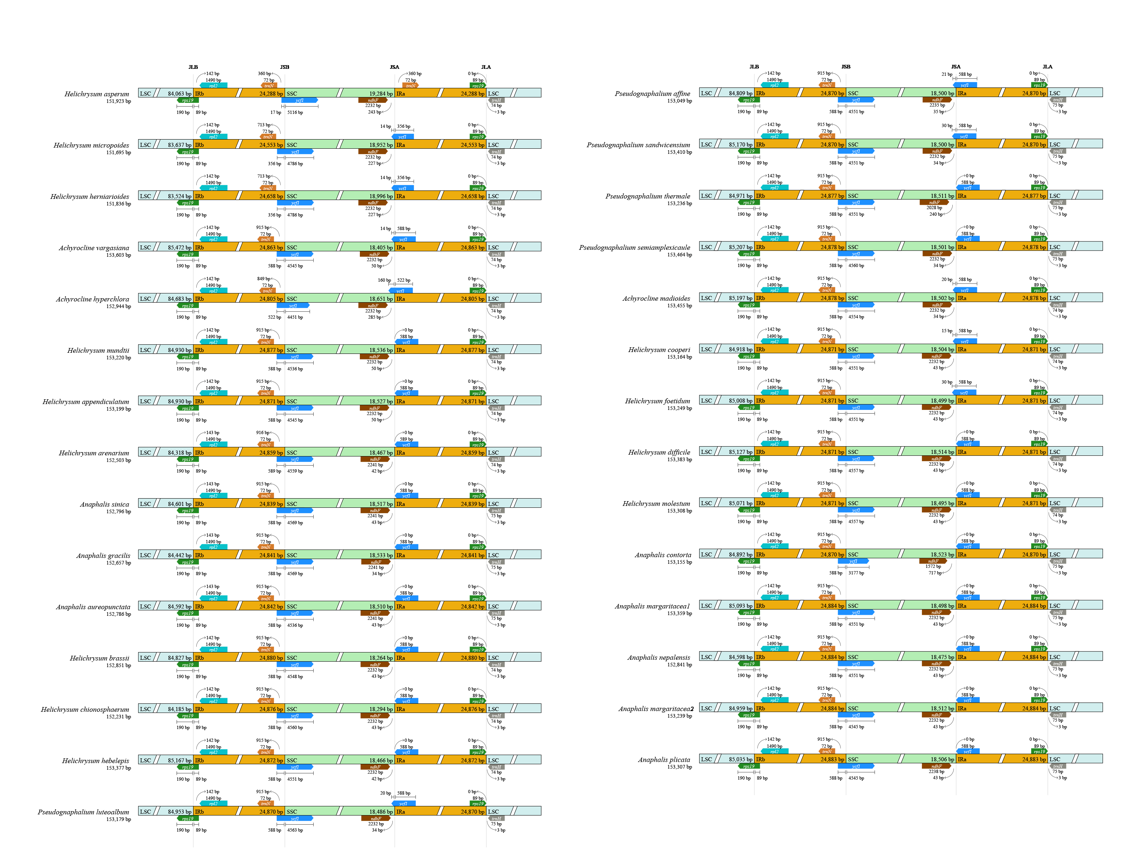


Figure S1. Gene order and junction sites for the HAP chloroplast genomes showing connection sites of LSC, IRB, SSC, and IRA. The T bars above or below the genes indicate the extent of their parts with their corresponding values in the base pair. The plotted genes and distances in the vicinity of the junction sites are the scaled projection of the genome. JLB (IRB/LSC), JSB (IRB/SSC), JSA (SSC/IRA), and JLA (IRA/ LSC) represent the junction sites between two adjacent regions in the genome.


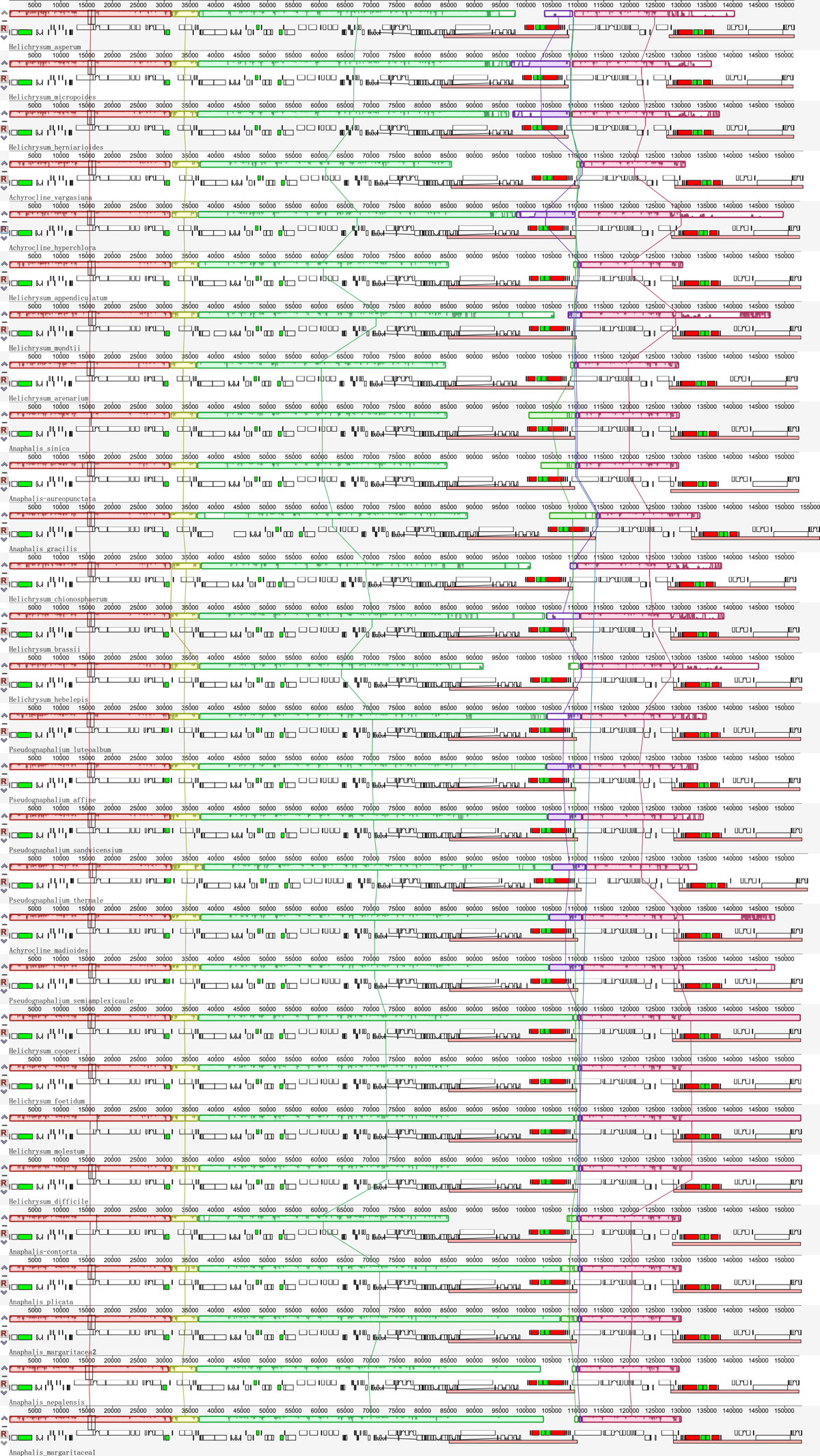


Figure S2. MAUVE alignment of the HAP chloroplast genomes. The *Helichrysum asperum* genome is shown at the top as the reference genome. Within each of the alignments, local collinear blocks are represented by blocks of the same color connected by lines.





Figure S3. A Bayesian tree based on the chloroplast genome sequences from the HAP and other taxa of Gnaphalieae. Numbers at nodes indicate Bayesian posterior probabilities (PP).


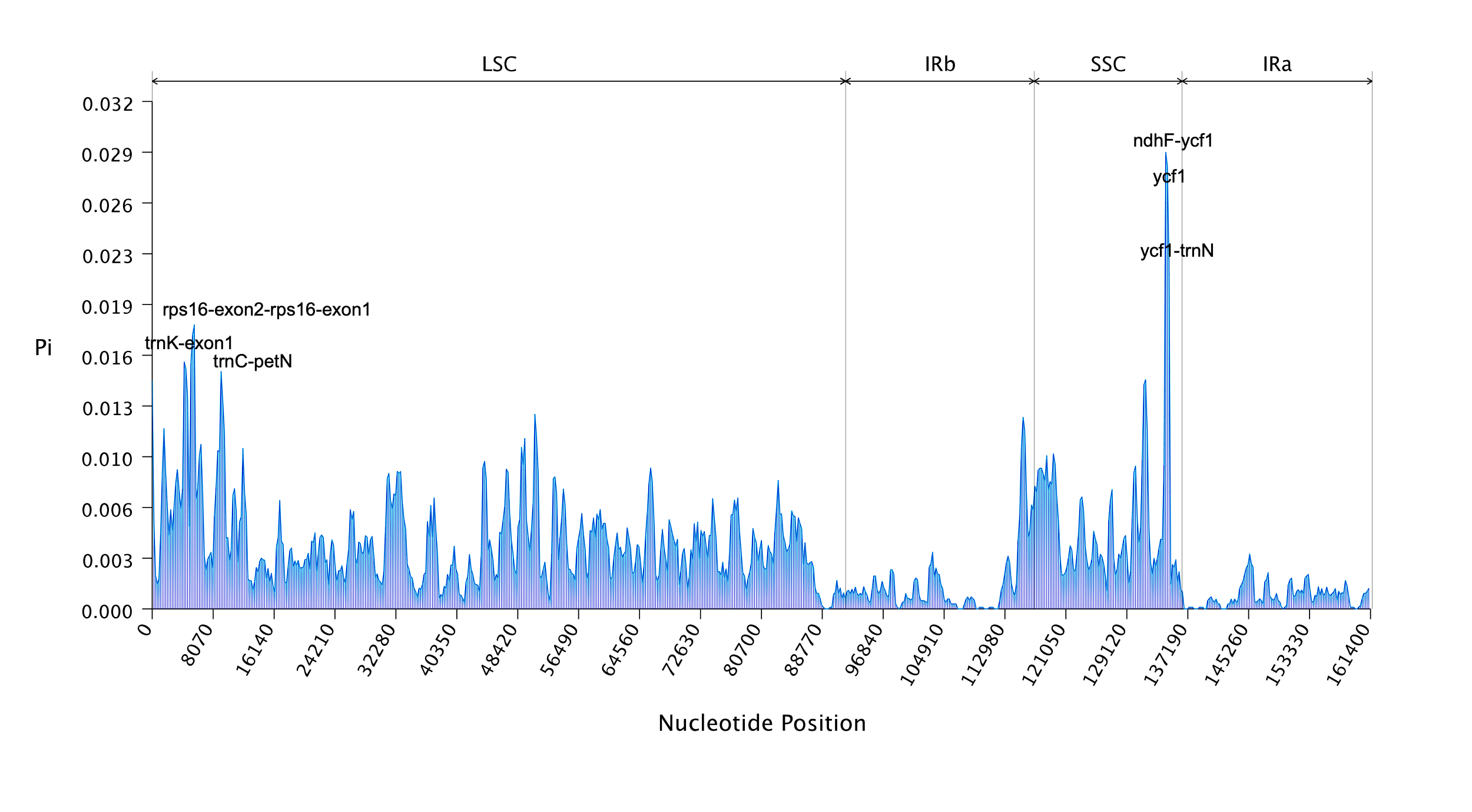


Figure S4. The nucleotide diversity values of the 29 chloroplast genomes from the HAP. X-axis: position of the window midpoint, Y-axis: nucleotide diversity within each window.


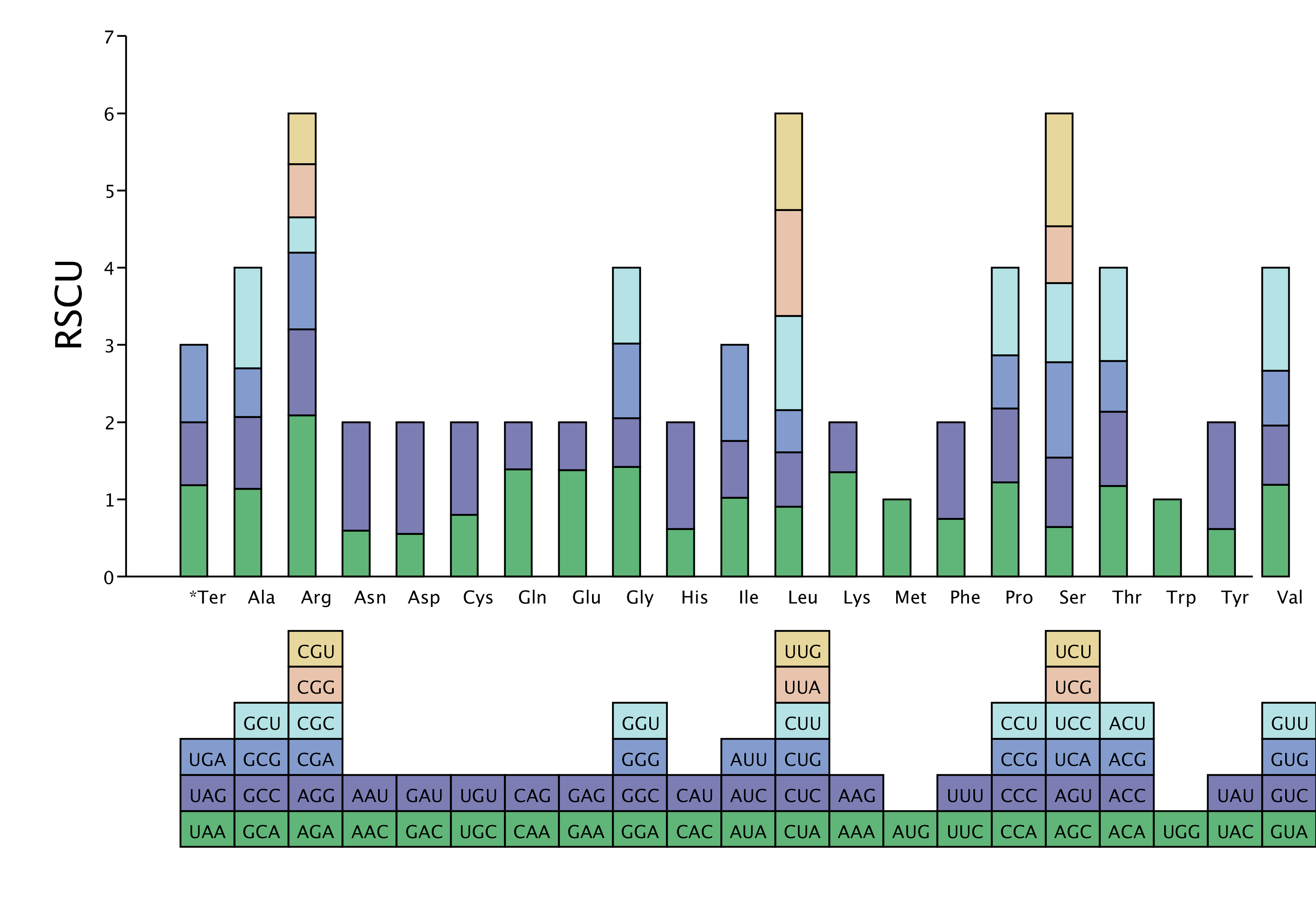


Figure S5. Amino acid frequencies of the chloroplast genomes of the HAP clade.


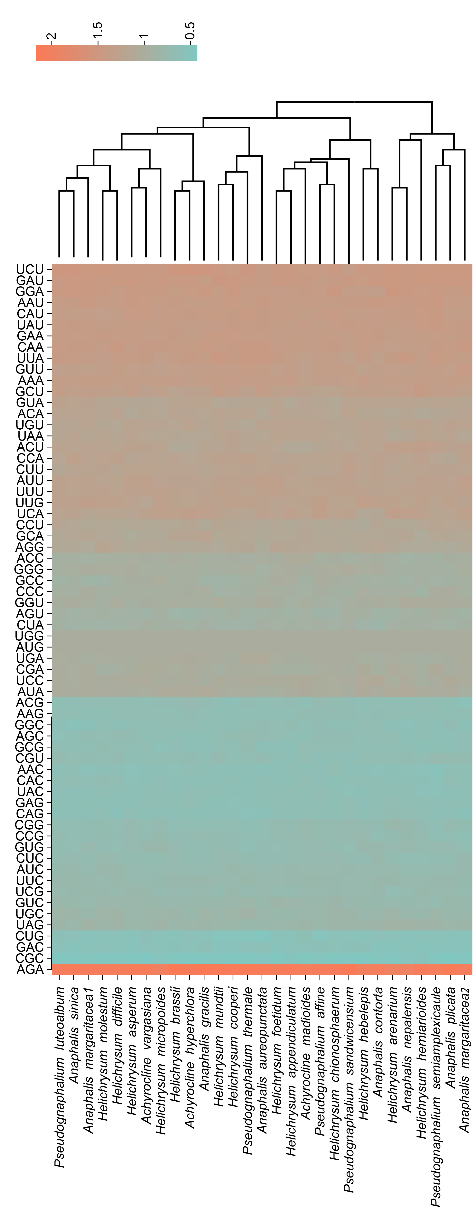


Figure S6. The heat map of RSCU clustering for the HAP plastomes. Color depth represents the Euclidean distance.
